# *In Vivo* Dioxin Favors Interleukin-22 Production by Human CD4+ T Cells in an Aryl Hydrocarbon Receptor (AhR)-Dependent Manner

**DOI:** 10.1371/journal.pone.0018741

**Published:** 2011-04-15

**Authors:** Nicolò Costantino Brembilla, Jean-Marie Ramirez, Rachel Chicheportiche, Olivier Sorg, Jean-Hilaire Saurat, Carlo Chizzolini

**Affiliations:** 1 Department of Immunology and Allergy, Swiss Centre for Applied Human Toxicology, University Hospital and School of Medicine, Geneva, Switzerland; 2 Department of Dermato-Toxicology, Swiss Centre for Applied Human Toxicology, University Hospital and School of Medicine, Geneva, Switzerland; French National Centre for Scientific Research, France

## Abstract

**Background:**

The transcription factor aryl hydrocarbon receptor (AhR) mediates the effects of a group of chemicals known as dioxins, ubiquitously present in our environment. However, it is poorly known how the *in vivo* exposure to these chemicals affects in humans the adaptive immune response. We therefore assessed the functional phenotype of T cells from an individual who developed a severe cutaneous and systemic syndrome after having been exposed to an extremely high dose of 2,3,7,8-tetrachlorodibenzo-*p*-dioxin (TCDD).

**Methodology/Principal Findings:**

T cells of the TCDD-exposed individual were studied for their capacity to produce cytokines in response to polyclonal and superantigenic stimulation, and for the expression of chemokine receptors involved in skin homing. The supernatants from T cells of the exposed individual contained a substantially increased amount of interleukin (IL)-22 but not of IL-17A, interferon (IFN)-γ or IL-10 when compared to nine healthy controls. *In vitro* experiments confirmed a direct, AhR-dependent, enhancing effect of TCDD on IL-22 production by CD4+ T cells. The increased production of IL-22 was not dependent on AhR occupancy by residual TCDD molecules, as demonstrated in competition experiments with the specific AhR antagonist CH-223191. In contrast, it was due to an increased frequency of IL-22 single producing cells accompanied by an increased percentage of cells expressing the skin-homing chemokine receptors CCR6 and CCR4, identified through a multiparameter flow cytometry approach. Of interest, the frequency of CD4+CD25^hi^FoxP3+ T regulatory cells was similar in the TCDD-exposed and healthy individuals.

**Conclusions/Significance:**

This case strongly supports the contention that human exposure to persistent AhR ligands *in vivo* induce a long-lasting effect on the human adaptive immune system and specifically polarizes CD4+ T cells to produce IL-22 and not other T cell cytokines with no effect on T regulatory cells.

## Introduction

2,3,7,8-Tetrachlorodibenzo-*p*-dioxin (TCDD) is the most potent member of a group of halogenated aromatic hydrocarbons, generally known as dioxins [1]. Dioxins are produced when organic material is burned in the presence of chlorine and are therefore widely implicated in many industrial as well as natural processes. Major sources of environmental dioxins include waste incinerators and steel industry as well as the use of herbicide and pesticide containing chlorophenols. Due to their high lipophilicity and poor metabolism, dioxins accumulate in lipid-rich tissues of animals and rapidly climb the food chain up to humans [2]. In addition, dioxins are presents in cigarette smoke. As a consequence, concentrations of dioxins are found in all humans, with higher levels commonly identified in persons living in industrialized countries.

TCDD, considered as the prototypical dioxin, has been shown to have pleiotropic biological effects at low doses in multiple animal species [3], [4]. The majority of TCDD effects are mediated via binding and activation of the intracellular aryl hydrocarbon receptor (AhR), as demonstrated by the loss of responsiveness to TCDD in AhR knockout mice [5]. The elevated toxicity of TCDD is caused by its extremely high affinity for AhR and its long half-life (5–10 years in humans [6]). Upon ligand binding AhR undergoes a conformational change and translocates into the nucleus, where it dimerizes with the AhR nuclear translocator (ARNT) and regulates, by binding to xenobiotic response elements (XRE), the expression of a variety of genes, including the xenobiotic metabolizing enzyme *CYP1A1* (cytochrome P450) [7]. In mice, AhR activation is reported to regulate T helper (Th) 17 and T regulatory (Treg) cell differentiation and to modulate immune responses to experimental induced encephalomyelitis in a ligand-dependent manner [8], [9], [10]. In addition, AhR has been shown to be crucial for interleukin (IL)-22 expression [10] and a regulatory mechanism for IL-22 production via a Notch-AhR axis has been identified [11]. We [12] and others [13] have demonstrated a role for AhR agonists, including TCDD, in promoting the *in vitro* production of IL-22 but not of IL-17 by human CD4 T cells. The possibility that in humans AhR stimulation could participate to the *in vitro* differentiation of IL-10-producing Treg cells has also been suggested [14].

IL-22 is a member of the IL-10 family of cytokines and signals via a receptor consisting of IL-22R and IL-10R2 subunits. IL-22 does not serve the communication between immune cells since cells of hematopoietic origin do not express IL-22R [15]. It mainly acts on epithelial cells of the gastrointestinal tract and the skin, where it promotes antimicrobial defense, protection against damage and regeneration [16]. However, its role in chronic inflammatory disorders may be either protective [17] or highly pathogenetic [18], [19]. T cells able to produce IL-22 in the absence of IL-17 and interferon (IFN)-γ, have been named Th22 cells and are enriched in cells expressing the skin-homing chemokine receptors CCR6, CCR4 and CCR10 while lacking CXCR3 [13], [20]. Th22 cells have been identified in the skin of individuals suffering of psoriasis and atopic dermatitis [21], [22], [23] and are thought to be important in skin immunosurveillance and immunopathology [13], [20], [21].

All the data on the effects of AhR ligands on human T cell subsets have been so far generated *in vitro* and their relevance to *in vivo* situations remains largely unknown. In this report, we extensively characterize the long-term immunological modifications induced by TCDD in one of the two ever reported cases of a human being who survived the *in vivo* exposure to an extremely high dose of the pure compound [24]. Our data indicate for the first time that *in vivo* exposure to TCDD induces a selective increase in the frequency of T cells producing IL-22 but neither IL-17, IL-10, nor IFN-γ, which preferentially express skin-homing chemokine receptors. These data strongly support the *in vivo* existence in humans of Th22 cells that depend on AhR for their expansion.

## Materials and Methods

### Patient

We obtained written approval from the patient to release peer-reviewed scientific information about his case. Our patient had been intoxicated by pure dioxin (TCDD) presumably in late 2004 at the age of 50. In early January 2005 he arrived under controlled conditions at the Geneva University Hospital, Switzerland, where we identified a TCDD concentration of 108,000 pg/g of lipid weight in his blood serum [24]. Similar levels were identified by an independent laboratory in a sample taken from the same patient in mid-December 2004 [25]. This concentration was more than 50,000 times the averaged TCDD in the general population (normal value: 10–20 pg/g of lipid weight) [26]. The patient was suffering from a severe skin disease, the historically called “chloracne”, consisting in what we call now “metabolizing acquired dioxin-induced skin hamartomas” (MADISH) to describe his skin condition [27]. All the experiments shown in this report were performed 4 years after the acute exposure to TCDD, when the patient had a TCDD concentration of 19,000 pg/g of lipid weight in his blood serum and no TCDD-related pathology was anymore clinically apparent. Nine sex and age (52±10 years) matched healthy members of the laboratory served as controls. CD4 and CD8 T cell frequencies in the peripheral blood of the TCDD-exposed individual were within the range of healthy controls (CD4+CD3+ T cells: 62.4% and 61±7% of living lymphocytes, respectively; CD3+CD8+ T cells: 25.7% and 18±7% of living lymphocytes, respectively). Permission to perform this investigation was granted by Comité departemental de médecine interne et médecine communautère des hôpitaux universitaires de Genève. Written informed consent according to the Helsinki declaration was obtained from each individual involved in this study.

### Reagents

Fetal calf serum (FCS), phorbol myristate acetate (PMA), β-mercaptoethanol, staphylococcal enterotoxin B (SEB) and brefeldin A were from Sigma Chemicals (St. Louis, MO); TCDD from Cambridge Isotope Laboratories (Andover, MA); ionomycin from Calbiochem (Merck KGaA, Darmstadt, Germany); RPMI 1640 medium, phosphate buffered saline (PBS), penicillin, streptomycin, L-glutamin, nonessential amino acids, sodium pyruvate from Life Technologies (Carlsbad, CA); human rIL-2 from Biogen (Zug, Switzerland); anti-IL-22-PE from R&D (Minneapolis, MN); anti-CD28 (CD28.2), anti-CD45RA-FITC, anti-CCR6-PercPCy5.5, anti-CCR4-PECy7, anti-CXCR3-APC, anti-CD4-PE-Cy5, anti-CD4-FITC, anti-CD4-APC-Cy7, anti-CD8-APC-Cy7 and anti-CD3-FITC from BD (Franklin Lakes, NJ); anti-IL-17A-FITC, anti-IL10-Alexa 488 and anti-IFN-γ-PE-Cy7 from Biolegend (San Diego, CA); anti-CD25-APC and anti-FoxP3-PE from eBioscience (San Diego, CA); anti-CD3 (OKT3) Ab from ATCC (Manassas, VA); CH-223191 from VWR (Dietikon, Switzerland).

### Cell culture

**Figure 1:**
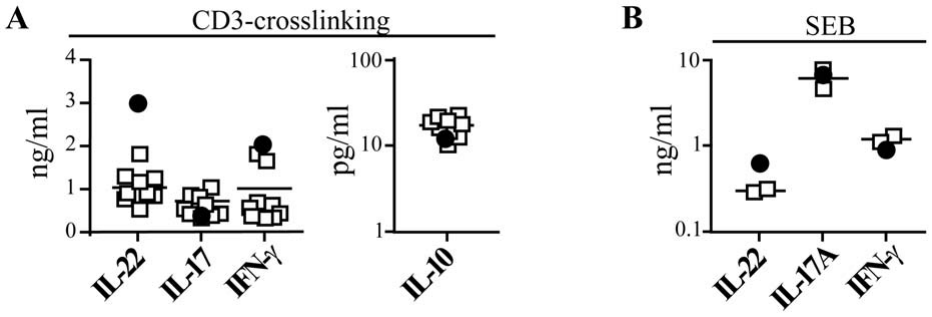
*In vivo* TCDD-exposure increases IL-22 but does not concomitantly affect IL-17A, IFN-γ and IL-10 production by PBMC. Cytokine levels were assessed at d 7 in supernatants of PBMC activated by CD3-crosslinking (**A**) or by Staphylococcal Enterotoxin B (SEB) (**B**). IL-2 (20 U/ml) was added 48h after culture initiation. Individual data for a single TCDD-exposed individual (full symbols) and 9 (CD3-crosslinking) or 2 (SEB stimulation) different healthy individuals (empty symbols) are shown.

Peripheral blood mononuclear cells (PBMC) were purified by Ficoll-paque Plus (GE Healthcare, Pittsburgh, PA) gradient centrifugation and frozen in liquid nitrogen until use or processed immediately (experiment in Fig. 1A). The cells from the TCDD-exposed individual were treated under the same experimental conditions and in parallel to that of healthy individuals in all experiments shown in this manuscript. PBMC were rested o/n in RPMI 1640 medium supplemented with 10% FCS (complete RPMI, cRPMI) as described [28] and then used in short term (up to 24 h) or long term (7 d) cultures. In short term cultures PBMC were activated for 4.5 h by PMA (50 ng/ml) and ionomycin (1 µM) or for 24 hours in flat-bottom 96-well plates in presence of coated anti-CD3 mAb (1 µg/ml) and soluble anti-CD28 mAb (1 µg/ml). When intracellular cytokine determination was performed, brefeldin A (2.5 µg/ml) was added after 1.5 and 3 hours from the beginning of the activation, respectively. In long-term activation experiments, PBMC were cultured at 1×10^6^ cells/ml in 24-well plates in cRPMI in the presence of soluble anti-CD3 Ab (0.1 µg/ml) or SEB (0.2 µg/ml). Culture medium was supplemented with IL-23 (10 ng/ml) and IL-2 (20 U/ml) was added 48 h after culture initiation. When used, TCDD (10 nM, unless otherwise specified) and CH-223191 (3 µM) were added at the beginning of the culture. At day 7 of culture, supernatants were collected and frozen until cytokine determination and the cells were harvested and activated for 4.5 h by PMA and ionomycin for FACS analysis.

### Flow cytometry and cytokine assays

Cell surface and intracellular staining were assessed by FACS analysis using FACSCanto (BD) and data were analyzed by FlowJo software 7.5 (Tree Star). For intracellular cytokine determination, cells were stained with anti-CD4-PE-Cy5 or anti-CD4-APC-Cy7 or anti-CD3-FITC mAbs, fixed and stained with anti-IL-17A-FITC or anti-IL-10-Alexa 488, anti-IL-22-PE, anti-IFN-γ-PE-Cy7 and anti-IL-4-APC mAbs using BD Cytofix/Cytoperm kit according to manufacturer’s instruction. To determine the frequency of Treg cells, PBMC were stained with anti-CD4-APC-Cy7, anti-CD45RA-FITC and anti-CD25-APC, fixed and stained with anti-FoxP3-PE using eBioscience Foxp3 Fixation/Permeabilization buffer according to manufacturer’s instruction. In chemokine receptor expression experiments, PBMC were stained with anti-CD4-APC-Cy7, anti-CD45RA-FITC, anti-CCR6-PercPCy5.5, anti-CCR10-PE, anti-CCR4-PECy7 and anti-CXCR3-APC following by FACS analysis.

IFN-γ, IL-22 and IL-10 were quantified in culture supernatants at day 7 of culture by ELISA (R&D Systems or for IL-10 Sanquin, Amsterdam, The Netherlands). IL-17 was quantified by ELISA (R&D) or by Luminex xMAPTM Technology using multiplex beads immunoassay (Fluorokine MAP Multiplex Human Cytokine Panel, R&D).

### Real-time quantitative PCR

Total RNA was extracted from resting PBMC using RNAesy micro kit (Quiagen, Hilden, Germany) and cDNA synthesized from 0.25 µg of total RNA using random hexamers and Superscript III reverse transcriptase (Invitrogen, Carlsbad, CA) according to manufacturer’s instructions. SYBR Green assays were performed on a SDS 7900 HT instrument (Applied Biosystems). Each reaction was performed in triplicates. Raw Ct values obtained with SDS 2.2.2 software (Applied Biosystems) were analyzed and the more stable housekeeping genes TBP (TATA-box-binding protein) and EEF1A1 (eukaryotic translation elongation factor 1 alpha 1) selected for normalization. All oligonucleotides were obtained from Quiagen: CYP1A1, QT00012341; TBP1, QT00000721; EEF1A1, QT01669934.

## Results and Discussion

CD4+ T cells characterized by the preferential production of IL-22 in the absence of IL-17 and IFN-γ have been recently described by us and others in the peripheral blood of healthy individuals [12], [13], [20]. These cells have been named Th22 cells. Cells with similar characteristics have been detected in the skin of individuals suffering of psoriasis and atopic dermatitis [21], [22]. In humans, AhR natural ligands such as the tryptophan photoproduct 6-formylindolo[3,2-b]carbazole (FICZ) and β-naphthoflavone as well as the synthetic ligand TCDD, have been shown to favor the *in vitro* polarization of naïve CD4+ T cells toward IL-22-single producing cells [12], [13]. However, whether previous *in vivo* exposure to an AhR-ligand in humans could favor the preferential outgrowth of a particular cell subset, thus affecting the adaptive T cell response, is unknown at present. To test this hypothesis, we determined the capacity of PBMC of a person surviving the exposure to an extremely high dose of the stable AhR ligand TCDD to produce cell subset discriminating cytokines upon CD3-crosslinking. When compared to those of 9 healthy individuals, the PBMC of the TCDD-exposed individual produced at base-line 3-fold higher levels of IL-22 but similar levels of IL-17A, IFN-γ and IL-10 (**Figure 1A**). Similarly, PBMC of the TCDD-intoxicated individual secreted higher amounts of IL-22 but not of IL-17A and IFN-γ following superantigen stimulation with Staphylococcal Enterotoxin B (SEB) (**Figure 1B**). SEB is a superantigen able to specifically activate a subset of T cells expressing the T cell receptors Vβ3, Vβ12, Vβ13.2, Vβ14, Vβ15, Vβ17 and Vβ20 chains [29]. Thus, persistent TCDD-exposure *in vivo* does not favor in humans the outgrowth of IL-17 producing Th17 cells, as observed in mice [9], [10], [30], [31], nor of IL-10 producing Tr1 cells, as observed *in vitro* in humans [8], [9], [14]. In contrast, it selectively favors the production of IL-22, induced in response to both mitogenic and superantigenic stimulation.

**Figure 2:**
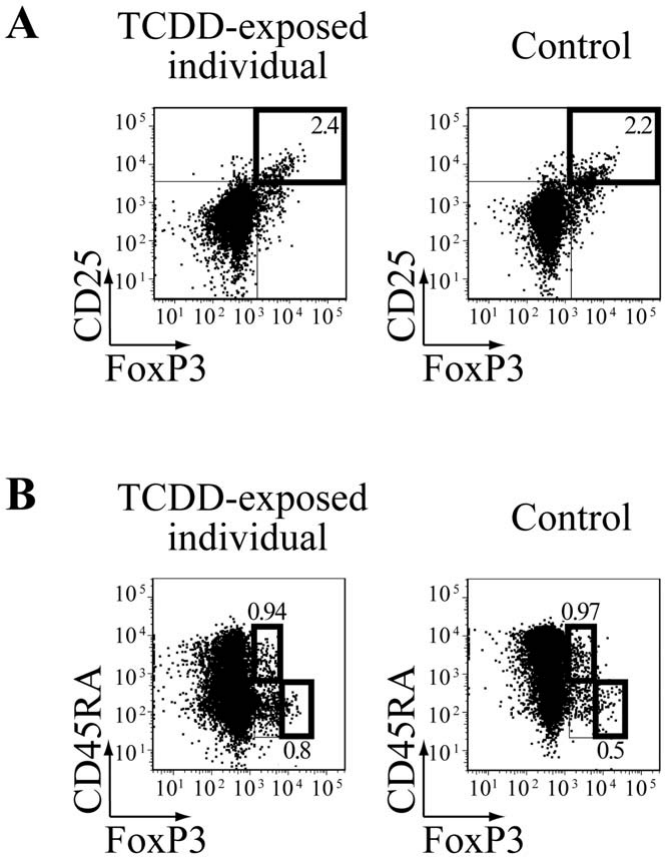
The levels of Treg cells are comparable in the peripheral blood of TCDD-exposed and healthy individuals. Surface staining of *ex-vivo* isolated PBMC from the TCDD-exposed and a representative healthy individual of five tested. Plots are gated on CD4+ T cells, numbers in plots indicate the percentage of CD25^hi^FoxP3+ Treg cells (**A**) or that of CD45RA+FoxP3^lo^ resting and CD45RA-FoxP3^hi^ activated Treg cells (**B**) in the CD4+ T cell fraction.

To further address the possible expansion of Treg cells upon exposure to TCDD as documented in mice [8], [9], we assessed the frequency of CD4+ T cells simultaneously expressing high levels of the IL-2 receptor subunit CD25 and the Treg-specific transcription factor FoxP3 in resting conditions [32]. We observed comparable levels of CD25^hi^FoxP3+ cells in the CD4+ T cell fraction of *ex-vivo* isolated PBMC from the TCDD-exposed and 5 healthy individuals (2.49% and 2.28±1.13% of CD4 T cells, respectively) (**Figure 2A**). Similarly, no difference were identified in the frequency of both resting (CD45RA+FoxP3^lo^: 0.94% and 1.65±0.92% of CD4 T cells, respectively) and activated (CD45RA-FoxP3^hi^: 0.8% and 0.74±0.25% of CD4 T cells, respectively) Treg cells, as defined by the expression of the phosphatase CD45RA and the transcription factor FoxP3 (**Figure 2B**) [32]. Thus, the frequency and subset composition of T cells with regulatory function was unaffected by TCDD exposure, although TCDD levels were still 10,000 times above normal values.

**Figure 3:**
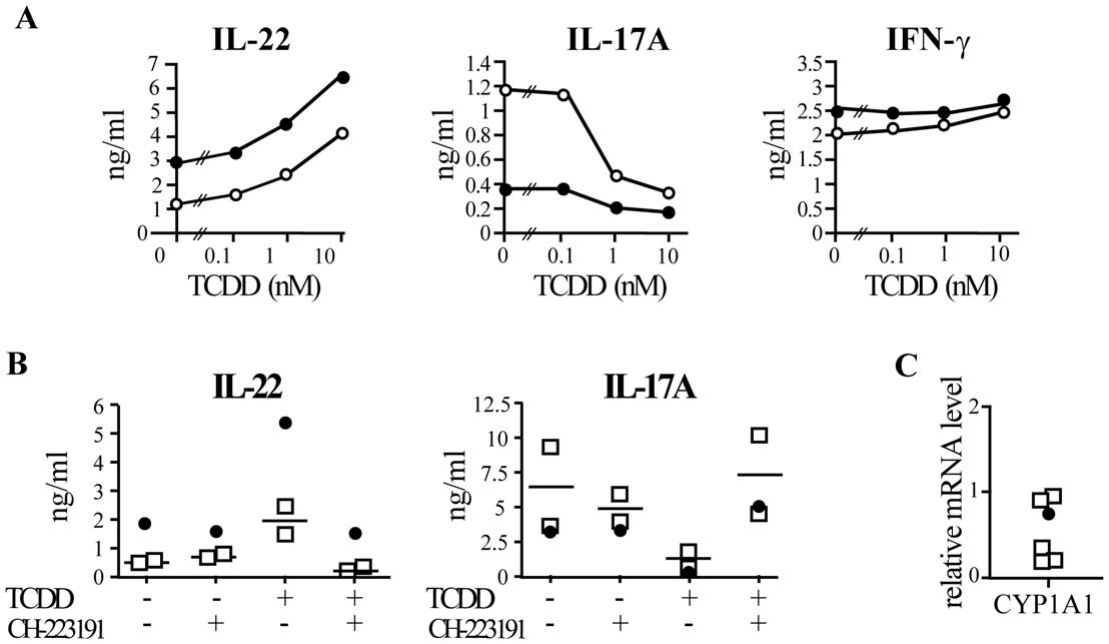
*In vitro* TCDD enhances IL-22 and down-regulates IL-17A production by PBMC in an AhR dependent manner. (**A**) PBMC were activated by CD3-crosslinking in the presence or absence of TCDD and the specific AhR inhibitor CH-223191. Cytokine levels were assessed in the supernatants harvested at d 7. Dose-dependent responses to TCDD in a single TCDD-exposed individual (full symbols) and one healthy individual (empty symbols). (**B**) Culture conditions as in A. Cytokine determination in a single TCDD-exposed individual (full symbols) and 2 healthy individuals (empty symbols) (**C**) mRNA levels of *CYP1A1* quantified by real-time PCR of resting PBMC from a single TCDD-exposed (full symbols) and five healthy individual (empty symbols). Expression levels have been normalized against the geometric mean of two house-keeping genes (EEF1A1 and TBP).

We next tested whether *in vitro* TCDD could modulate the production of IL-22, IL-17A and IFN-γ by PBMC activated upon CD3-crosslinking. We found that TCDD dose-dependently increased further the production of IL-22 in the TCDD-exposed individual and, as expected, boosted IL-22 production in controls. This increase was specific as far as IFN-γ was not affected while IL-17A production decreased in the presence of TCDD (**Figure 3A**). Thus, exposure to TCDD in our patient did not alter the ability of his lymphocytes to respond to TCDD stimulation and did not result in lymphocyte exhaustion. Together, these data corroborate and expand previous observations demonstrating a direct ability of TCDD to modulate the production of IL-22 cytokine in short-term culture *in vitro* [12].

**Figure 4:**
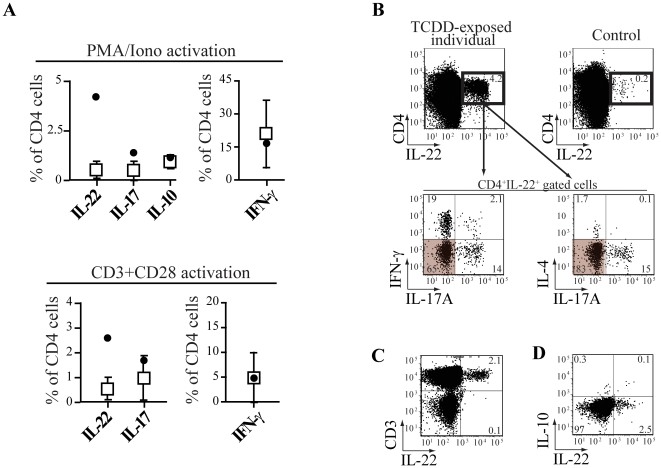
*In vivo* TCDD-exposure favors the expansion of Th22 cells. (**A**) Intracellular staining of *ex-vivo* isolated PBMC from a TCDD-exposed (full symbols) and healthy individuals (empty symbols) upon activation by PMA/Ionomycin for 4.5 h (n = 5) or CD3/CD28 crosslinking for 24 h (n = 9). (**B, C, D**) Representative FACS plots of cells activated by PMA/Ionomycin from the TCDD-exposed and a control individual (upper right panel in B) after gating on the forward and side scatter of viable PBMC (**B, C**) or on CD4+ cells (**D**). Numbers in plots indicate the percentage of cells in each quadrant.

TCDD is thought to form a long-lived and stable complex with AhR [7]. Thus, the higher amount of IL-22 produced by PBMC of the TCDD-exposed individual in the absence of exogenously added TCDD could result from the persistent action of the ligand on a stable number of cells. Alternatively, it could be explained by an increased frequency of IL-22-producing cells previously primed *in vivo* under the influence of TCDD. To discriminate between these two hypotheses, we cultured the PBMC in the presence of the high-affinity AhR-antagonist CH-223191. If residual TCDD was binding AhR in the TCDD-exposed individual, the AhR antagonist would reduce the amount of IL-22 produced upon T cell activation by displacing at least in part the bound TCDD, otherwise no effects would be observed. The presence of the AhR antagonist CH-223191 in the culture in the absence of TCDD did not decrease the basal level of IL-22 produced by the TCDD-exposed subject to the one observed in healthy individuals (**Figure 3B**). However, the specific AhR antagonist completely reversed the enhanced IL-22 and decreased IL-17A production observed when exogenous TCCD was added to the cultures in both the TCDD-exposed and healthy individuals (**Figure 3B**). To confirm these findings, we assessed the mRNA levels of the AhR-target gene *CYP1A1*, which is readily up-regulated by TCDD ligation [7]. In agreement with the lack of inhibition by CH-223191 in basal conditions, which suggests no occupancy of TCDD binding sequences, no differences were observed in the transcription level of CYP1A1 in resting PBMC from the TCDD-exposed and control individuals (**Figure 3C**). These data demonstrate the involvement of AhR in mediating dioxin effects and, most importantly, suggest that the AhR receptor was not anymore occupied in the TCDD-exposed individual at the time of our investigation. Thus, the increased production of IL-22 could be explained by an increased frequency of cells producing IL-22, which accumulated with time while responding to novel antigenic challenges under the polarizing effect exerted by TCDD. To test this hypothesis we assessed the frequency of *ex vivo*-detectable IL-22-secreting CD4+ cells in PBMC from the TCDD-exposed and healthy individuals. We observed that the frequency of CD4+ cells producing IL-22 was at least 3-fold higher in the TCDD-exposed individual compared to controls. By contrast, the frequency of cells producing IL-17A, IL-10 and IFN-γ was similar (**Figure 4A**). The increase in IL-22 producing cells was reproducible in short term cultures across a variety of activation stimuli, which included TCR-dependent (CD3 and CD28 cross-linking) and TCR-independent (PMA and ionomycin) signaling (**Figure 4A and B**). In the CD4+ compartment, IL-22 is mainly produced by CD3+ T cells and to a minor extent by CD3- cells including lymphoid tissue inducer (LTi) cells [33]. In the TCDD exposed individual, all IL-22 producing cells were CD3+, thus indicating that the source of IL-22 was CD4+ T cells (**Figure 4B and C**). Of interest, multiparameter flow cytometry analysis revealed that the majority of the IL-22+ cells in the TCDD-exposed individual did not concomitantly produce IL-17A, IL-4, IFN-γ (**Figure 4B**) and IL-10 (**Figure 4D**). Altogether, these data strongly indicate that *in vivo* exposure to TCDD favors the expansion of CD4+ T cells having the functional properties of Th22 cells.

**Figure 5:**
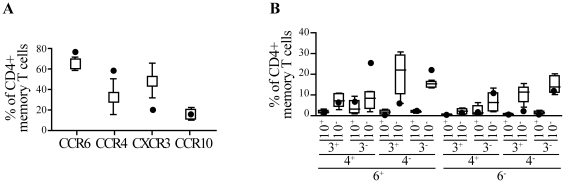
Increased frequency of CD4+ memory T cells expressing the skin-homing receptors CCR6+ and CCR4+ in an individual exposed *in vivo* to high levels of TCDD. Surface chemokine receptor expression on CD4+CD45RA- memory T cells from *ex-vivo* isolated PBMC in a TCDD-exposed (full symbols) or 5 healthy individuals (empty symbols). (**A**) Mean ± SD of cells expressing CCR6, CCR4, CXCR3 and CCR10. (**B**) Percentage of cells expressing various combinations of CCR6, CCR4, CXCR3 and CCR10. Data from the 5 healthy individuals are expressed as box plots. Box plots were automatically generated using GraphPad Prism version 4.00 for Windows (GraphPad Software). The box represents values between 25th and 75th percentile with a line at the median (50th percentile). The whiskers extend above and below the box to show the highest and the lowest values. 6, 4, 3 and 10 in panel B denote CCR6, CCR4, CXCR3 and CCR10, respectively.

Since the exposure to dioxin resulted in severe skin manifestations, we tested whether the pattern of chemokine receptors associated with skin homing expressed in CD4+ T cells was skewed in the TCDD-exposed individual [34], [35], [36], [37], [38]. We found that the frequency of CCR6+ and CCR4+ cells in the memory CD4 T cell compartment was higher in the TCDD-exposed individual than in controls, while the frequency of CXCR3+ cells was lower (**Figure 5A**). This is remarkable, since Th22 cells were shown to be enriched in CD4+ T cells expressing CCR6, CCR4 and CCR10 in absence of CXCR3 [13], [20]. A detailed multiparametric analysis performed at a single cell level, revealed that in the TCDD-exposed individual there was a substantial three-fold increase in the frequency of CD4+ memory T cells with the CCR6+CCR4+CXCR3-CCR10- phenotype and a modest increase in the CCR6+CCR4-CXCR3-CCR10- subset when compared to healthy controls (**Figure 5B**). No differences were identified in the CCR6- compartment, thus stressing the specificity of our finding. It is noteworthy that we did not observe a concomitant preferential expression of CCR10 in the TCDD-exposed individual as observed by others in Th22 cells [13], [20]. This may reflect a specific characteristic of T cell producing IL-22 under the influence of TCDD.

It is tempting to speculate that Th22 may have contributed to some extent to the pathogenesis of the dioxin induced skin lesions [27]. Th22 cells have been shown to be involved in skin disease such as psoriasis and atopic dermatitis [21], [22], [23] which molecular pathology is very different from the dioxin-induced skin lesions, historically called “chloracne” and now defined as “metabolizing acquired dioxin induced skin hamartomas” which primarily involve sebaceous glands [27]. Further studies are needed to analyze the putative effect, if any, of IL-22 in sebaceous gland pathology. However, It is also possible that the skewed pattern of chemokine receptor expression in the TCDD-exposed individual was only the consequence of the expansion of Th22 cells taking place with time.

In conclusion, we present here strong evidence indicating that prolonged exposure *in vivo* to high dose of TCDD induces a profound, long-lasting, perturbation of the adaptive immune system and specifically polarizes CD4+ T cells to produce IL-22 but not other T cell cytokines in an AhR-dependent manner. The best model explaining our findings suggest that antigenic exposure taking place under the influence of TCDD polarizes T cells to the Th22 subset. While historically relevant environmental disasters [39], [40], [41] caused people exposure to a mix of toxic agent including TCDD, the case here discussed represents a unique opportunity for investigating the effect of pure dioxin on human T cells. TCDD is one of the major environmental pollutants, present in food and in cigarette smoke. While current doses are largely below those observed in the index case, our observation helps to better understand the effect of dioxin on the human immune system.
